# Palliative gastrectomy or gastrojejunostomy for symptomatic stage IV gastric cancer: a dual center, retrospective, propensity matched cohort study

**DOI:** 10.3389/fonc.2026.1892394

**Published:** 2026-07-22

**Authors:** Xiaojie Zhang, Hu Ren, Lin Zhang, Zongze Li, Shengnan Zhou, Yingtai Chen, Dongbing Zhao, Lei Zhou

**Affiliations:** 1 Gastrointestinal Surgery Department, China-Japan Friendship Hospital, Beijing, China; 2 Department of Pancreatic and Gastric Surgical Oncology, National Cancer Center/National Clinical Research for Cancer/Cancer Hospital, Chinese Academy of Medical Sciences and Peking Union Medical College, Beijing, China; 3 Department of General Surgery, Beijing Tiantan Hospital, Capital Medical University, Beijing, China

**Keywords:** gastrectomy, gastric cance, gastrojejunostomy, stage IV, survival

## Abstract

**Background:**

Palliative surgery is considered to release symptoms for symptomatic stage IV gastric cancer (GC) patients. However, whether palliative gastrectomy or gastrojejunostomy should be conducted was still controversial. Therefore, the current study aims to compare the long-term prognosis of palliative gastrectomy and gastrojejunostomy for symptomatic stage IV GC patients.

**Methods:**

Symptomatic stage IV GC patients in the China-Japan Friendship Hospital and China National Cancer Center between January 2000 to December 2023 were retrospectively reviewed. The short-term safety and long-term survival outcomes were compared between palliative gastrectomy and gastrojejunostomy for symptomatic stage IV GC patients. A 1:1 propensity score matching (PSM) was performed to balance covariates between groups.

**Results:**

In total, 2705 gastric cancer with stage IV were retrieved and finally 197 individuals were therefore enrolled in the study based on the search and inclusion and exclusion criteria. Compared with palliative gastrectomy group, palliative gastrojejunostomy group had shorter operation time (*P* < 0.001) and lower proportion of intraoperative blood transfusion (37 [32.174%] vs. 9 [14.516%], *P* = 0.011). While no significant difference was observed about postoperative complications between the two groups. Multivariate survival analysis demonstrated similar prognosis for symptomatic stage IV GC patients who underwent palliative gastrectomy or palliative gastrojejunostomy before (HR: 1.215, 95%CI: 0.859~1.717, *P* = 0.270) and after (HR: 0.756, 95%CI: 0.526~1.087, *P* = 0.131) applying PSM.

**Conclusions:**

Palliative gastrectomy might provide no survival benefit compared with Gastrojejunostomy for symptomatic stage IV gastric cancer. However, further multicenter, prospective studies with a larger sample size were needed to verify these findings.

## 1 Background

Gastric cancer (GC) is one of the most common malignancies with an incidence of more than 1 million newly diagnosed cases (1). Unfortunately, a significant proportion of patients are initially diagnosed with metastasis diseases and, therefore, have remarkably poor prognosis (2). Notably, several stage IV GC patients were presented with bleeding or obstruction. Palliative surgery is considered to release symptoms for symptomatic stage IV GC patients.

However, whether palliative gastrectomy or gastrojejunostomy should be conducted was still controversial. Several previous studies have demonstrated that palliative gastrectomy offers no survival benefit over gastrojejunostomy for symptomatic stage IV GC patients (3–6). However, the evidence was largely inconclusive due to the small sample size. On the contrary, a recent retrospective cohort study conducted by Hiroshima Surgical study group of Clinical Oncology (HiSCO) suggest that palliative gastrectomy may improve the median survival time for symptomatic stage IV GC patients (22.0 months vs. 13.3 months) (7). Moreover, evidence from National Cancer Database also suggest that palliative gastrectomy for metastasis GC patients confers significant survival benefit (8). A recent randomized controlled clinical study on patients with malignant gastric outlet obstruction showed that endoscopic ultrasound-guided gastrojejunostomy was superior to traditional surgical gastrojejunostomy in both clinical efficacy and cost-effectiveness (9). The study data indicated that ultrasound-guided gastrojejunostomy could help patients resume oral feeding more quickly (median time 2 days), significantly shorten the hospital stay (median 3 days), reduce medical costs (average savings of $17,503), and better improve the quality of life of patients, without increasing the risk of the procedure (9). However, endoscopic ultrasound-guided gastrojejunostomy usually requires that the patient’s tumor be small enough to ensure that there is sufficient normal stomach wall for the endoscopic surgical procedure.

In view of this, the primary purpose of the current study was to compare the long-term survival between palliative gastrectomy and gastrojejunostomy for symptomatic stage IV GC patients. Thus, providing additional evidence to guide clinical practice.

## 2 Methods

### 2.1 Patients and study design

This was an observational, dual-center, retrospective cohort study. Primary-metastatic GC patients treated in China-Japan Friendship Hospital and China National Cancer Center between January 2000 to December 2023 were retrospectively reviewed. The main inclusion criteria were: (i) Patients who were pathologically confirmed as primary gastric cancer; (ii) Patients who were confirmed with distant metastasis disease through preoperative imaging examination or abdominal exploratory surgery; (iii) Patients with significant bleeding or obstruction symptoms; (iv) Patients who can tolerate surgical treatment after preoperative assessment; (v) Patients who underwent palliative gastrectomy or gastrojejunostomy; (vi) Informed consent for surgical operation were obtained from the patients. The main exclusion criteria were: (i) Patients who have only undergone abdominal exploratory surgery; (ii) Patients who underwent gastrointestinal stent implantation or gastrointestinal fistula creation surgery; (iii) Patients who suffered postoperative recurrence and metastasis after radical gastrectomy; (iv) Patients with other malignant tumor types; (v) Patients with incomplete important clinicopathological and prognostic information; (vi) To balance the data from the two centers, we excluded the patients who received cytoreductive surgery (CRS) and hyperthermic intraperitoneal (HIPEC) treatment. As this was a retrospective observational study, it was exempted from review by the Ethics Committees.

### 2.2 Surgical procedure

All the patients underwent abdominal exploratory surgery first to identify intraperitoneal metastases. After doctors informed the patients of the advantages and disadvantages of palliative gastrectomy and gastrojejunostomy, the surgical procedure was selected after communication between the patient and the medical team. According to the location of the tumor, the options for gastrectomy include distal subtotal gastrectomy and total gastrectomy. Methods of anastomosis included Billroth-I, Billroth-II, and Roux-en-Y reconstruction.

### 2.3 Follow-up and outcomes

The postoperative follow-up was performed through outpatient clinical visits, telephone contact, and death registries. Last follow-up was performed in August 30^th^, 2025. The median and interquartile range (IQR) follow-up time were 32 months, 26 months (IQR1), and 38 months (IQR3), respectively. The main long-term outcome was overall survival time (OS), which was defined as the time from surgery to the death or last follow-up. The second outcome was perioperative safety, which include operative time, hospital time, ICU treatment, blood transfusion, postoperative complications, and postoperative death.

### 2.4 Statistical analysis

All continuous variables were expressed by mean and standard deviation (SD), and *t*-tests were used for comparison between groups. All categorical variables were expressed by frequency and percentage, and comparison between groups was performed by Chi-square test and Fisher’s exact test. Survival curves were plotted using the Kaplan-Meier method and compared using the log-rank method. The Cox regression model was used for survival analysis, in which variables with univariate *P*-values less than 0.1 were included in the multivariate model. Thereafter, a propensity score matching (PSM) procedure was subsequently performed in a 1:1 ratio for gender, tumor location, symptoms, and palliative chemotherapy, with a caliper width of 0.2. All statistical analyses were conducted using SPSS software (SPSS Inc., Chicago, IL, USA, version 22.0) and R software (version 4.1.2).

## 3 Results

### 3.1 Baseline characteristics

**Figure 1:**
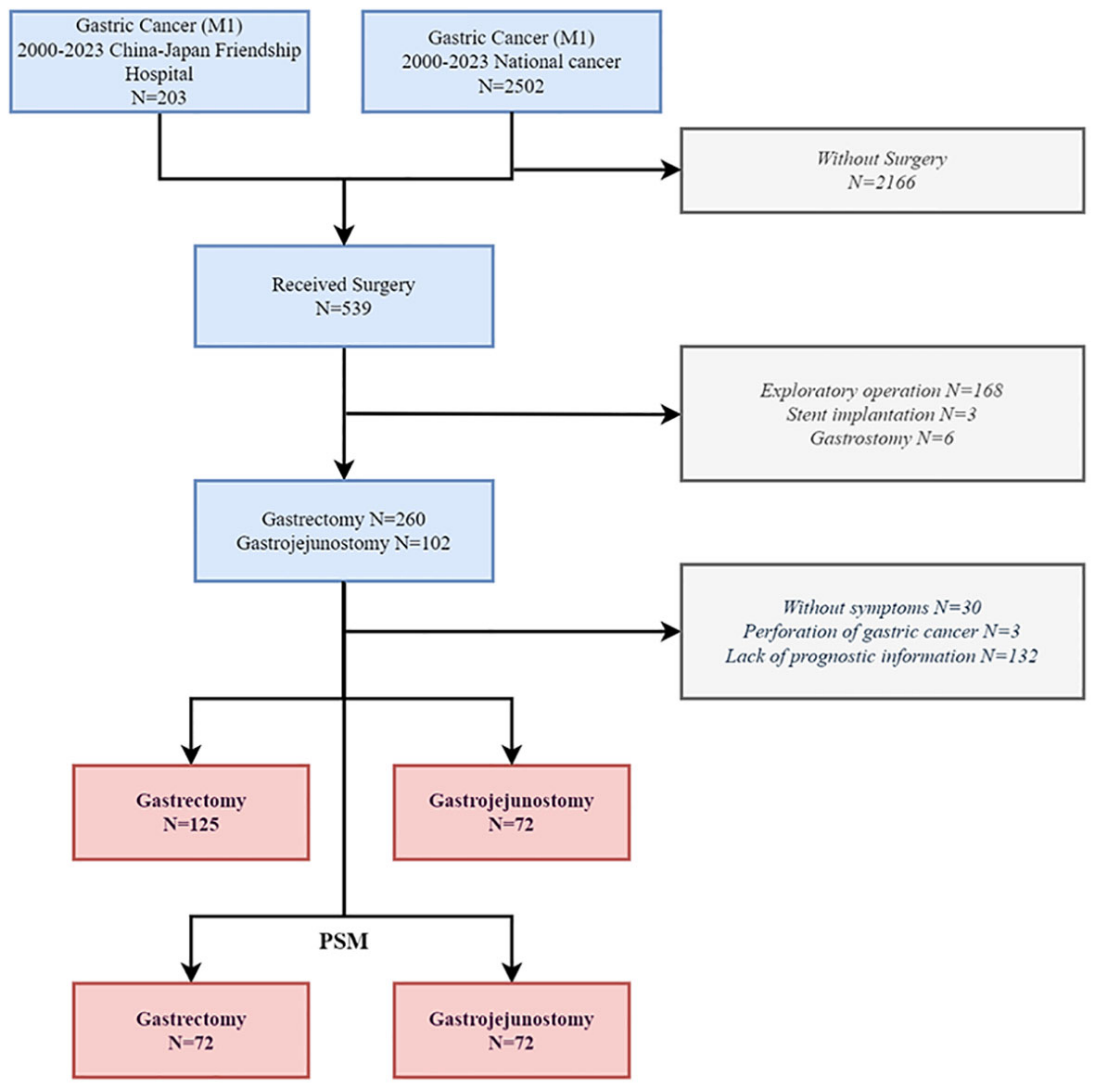
Flowchart of patient inclusion and exclusion criteria.

In total, 2705 gastric cancer with stage IV were retrieved and finally 197 individuals were therefore enrolled in the study based on the search and inclusion and exclusion criteria, among whom 125 received palliative gastrectomy and 72 received palliative gastrojejunostomy. Reasons for screening failures are detailed in **Figure 1**. Detailed baseline characteristics were presented in the **Table 1**. At baseline, there was no significant difference in age, body mass index (BMI) between gastrectomy group and gastrojejunostomy group. However, most symptomatic stage IV patients who receive palliative surgery had distal gastric cancer (156 [79.19%]). Moreover, most patients had had an open laparotomy (185 [93.91%]). From the perspective of surgical choice, patients with hemorrhage or patients with proximal gastric cancer are easier to choose palliative gastrectomy. For all the patients, the most common metastasis sites were peritoneal metastasis (145 [73.60%]), liver metastasis (33 [16.75%]), and ovary metastasis (17 [8.63%]). A total of 23 patients experienced multi-organ metastasis. Among them, 20 patients in the gastrectomy group, while 3 patients in the gastrojejunostomy group. The detailed metastatic sites were recorded in **Supplementary Table 1**.

**Table 1:** The baseline characteristics of symptomatic stage IV gastric cancer patients who underwent palliative gastrectomy or gastrojejunostomy.

Variables	Total (n=197)	Gastrectomy (n=125)	Gastrojejunostomy (n=72)	*P*-value
Age (mean ± SD)	56.015 ± 11.488	55.584 ± 11.652	56.764 ± 11.160	0.490
BMI (mean ± SD)	22.657 ± 3.415	22.705 ± 3.427	22.570 ± 3.389	0.795
Gender, n (%)				0.055
Male	131 (66.497)	77 (61.600)	54 (75.000)	
Female	66 (33.503)	48 (38.400)	18 (25.000)	
Tumor location, n(%)				0.015
Proximal	34 (17.259)	29 (23.200)	5 (6.944)	
Distal	156 (79.188)	92 (73.600)	64 (88.889)	
Total	7 (3.553)	4 (3.200)	3 (4.167)	
Smoking history, n(%)				0.450
Yes	64 (32.487)	43 (34.400)	21 (29.167)	
No	133 (67.513)	82 (65.600)	51 (70.833)	
Drinking history, n(%)				0.355
Yes	57 (28.934)	39 (31.200)	18 (25.000)	
No	140 (71.066)	86 (68.800)	54 (75.000)	
Symptoms, n(%)				0.006
Bleeding	19 (9.645)	16 (12.800)	3 (4.167)	
Obstruction	178 (90.355)	109 (87.200)	69 (95.833)	
Surgical approach, n(%)				0.140
Open	185 (93.909)	115 (92.000)	70 (97.222)	
Laproscope	12 (6.091)	10 (8.000)	2 (2.778)	
Surgical margins, n(%)				nan
Negative	114 (91.200)	114 (91.200)	nan	
Positive	11 (8.800)	11 (8.800)	nan	
Gastric stump cancer, n(%)				0.907
Yes	3 (1.523)	2 (1.600)	1 (1.389)	
No	194 (98.477)	123 (98.400)	71 (98.611)	
Linitis plastica, n(%)				0.140
Yes	12 (6.091)	10 (8.000)	2 (2.778)	
No	185 (93.909)	115 (92.000)	70 (97.222)	
Differentiation, n(%)				nan
Poor	102 (51.777)	95 (76.000)	7 (9.722)	
Moderate	16 (8.122)	15 (12.000)	1 (1.389)	
Well	1 (0.508)	0 (0.000)	1 (1.389)	
Unknown	78 (39.594)	15 (12.000)	63 (87.500)	
Lauren classification, n(%)				<0.001
Intestinal type	19 (9.645)	18 (14.400)	1 (1.389)	
Diffuse type	34 (17.259)	31 (24.800)	3 (4.167)	
Mixed type	17 (8.629)	11 (8.800)	6 (8.333)	
Unknown	127 (64.467)	65 (52.000)	62 (86.111)	
Palliative chemotherapy, n(%)				0.001
Yes	147 (74.619)	83 (66.400)	64 (88.889)	
No	5 (2.538)	3 (2.400)	2 (2.778)	
Unknown	45 (22.843)	39 (31.200)	6 (8.333)	

### 3.2 Short-term outcomes

Safety was one of the most critical factors when considering surgical selection. Compared with palliative gastrectomy group, palliative gastrojejunostomy group had shorter operation time (200.00 vs. 120.00, *P* < 0.001) and lower proportion of intraoperative blood transfusion (37 [32.17%] vs. 9 [14.52%], *P* = 0.011). However, overall incidence of postoperative complications did not differ significantly between the two groups. The most common postoperative complications were abdominal Infection, anastomotic fistula, and wound infection. In addition, 1 patient died perioperatively in the palliative gastrectomy group, while no perioperative death occurred in the palliative gastrojejunostomy group. The details were available in the **Table 2**.

**Table 2:** Comparing the short-term outcomes between palliative gastrectomy group and palliative gastrojejunostomy group.

Variables	Total (n=197)	Gastrectomy (n=125)	Gastrojejunostomy (n=72)	*P*-value
Hospital stays/days, median [IQR]	18.000[15.000,22.000]	18.000[16.000,22.000]	18.000[15.000,22.000]	0.367
Operative time/min, median [IQR]	180.000[130.000,225.000]	200.000[170.000,240.000]	120.000[90.000,155.000]	<0.001
Intraoperative blood transfusion, n(%)				0.011
Yes	46 (25.989)	37 (32.174)	9 (14.516)	
No	131 (74.011)	78 (67.826)	53 (85.484)	
ICU, n(%)				0.180
No	184 (93.401)	119 (95.200)	65 (90.278)	
Yes	13 (6.599)	6 (4.800)	7 (9.722)	
Perioperative death, n(%)				nan
No	193 (99.485)	124 (99.200)	69 (100.000)	
Yes	1 (0.515)	1 (0.800)	0 (0.000)	
Postoperative complications, n(%)				0.704
No	178 (91.282)	113 (91.870)	65 (90.278)	
Yes	17 (8.718)	10 (8.130)	7 (9.722)	
Anastomotic fistula	6 (3.046)	5 (4.000)	1 (1.389)	
Bleeding	4 (2.030)	2 (1.600)	2 (2.778)	
Abdominal Infection	8 (4.061)	6 (4.800)	2 (2.778)	
Wound infection	5 (2.538)	3 (2.400)	2 (2.778)	
Others	3 (1.523)	1 (0.800)	2 (2.778)	

### 3.3 Long-term survival analysis

**Figure 2:**
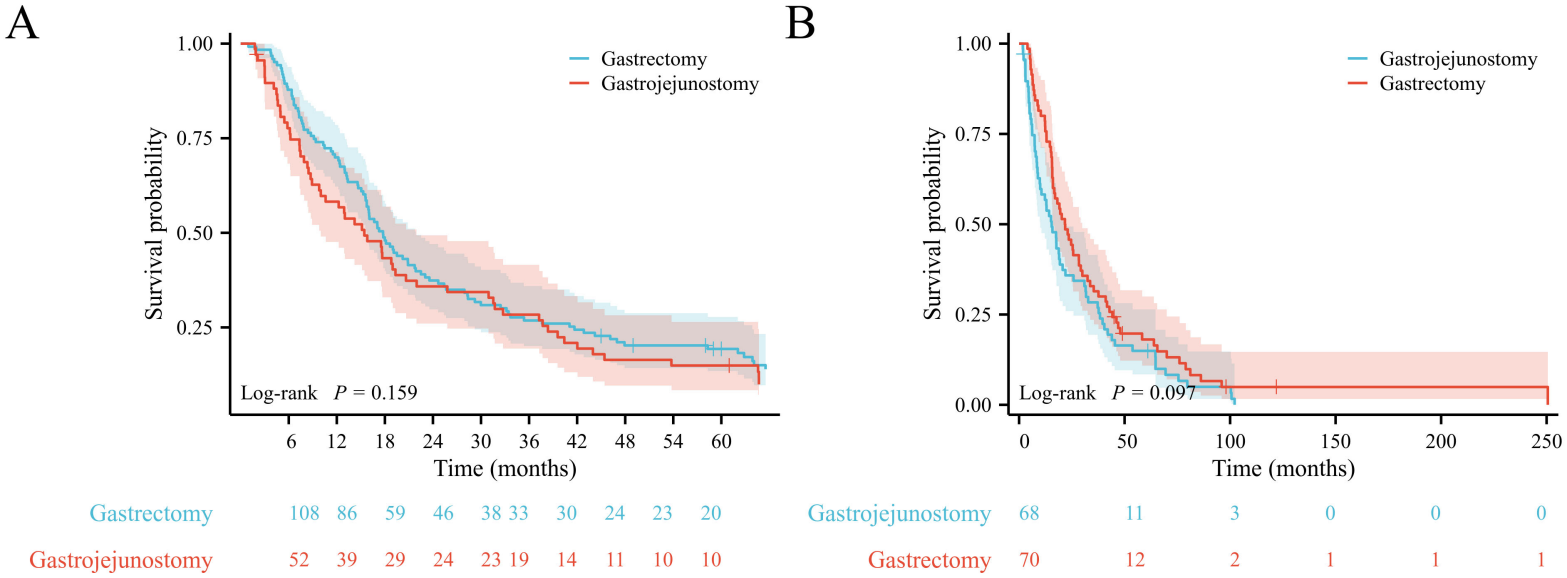
The survive curves of the palliative gastrectomy group and palliative gastrojejunostomy group before **(A)** and after **(B)** PSM.

The 1-year, 3-year, and 5-year OS rate for the total patients were 65.8%, 27.4%, and 17.8%, respectively. The 1-year, 2-year, and 5-year OS rate for the palliative gastrectomy group and palliative gastrojejunostomy group were 69.9% vs. 58.2%, 26.8% vs. 28.4%, 18.2% vs. 14.9%, respectively. The Kaplan–Meier survival curve analysis demonstrated similar prognosis for symptomatic stage IV GC patients who underwent palliative gastrectomy or palliative gastrojejunostomy (**Figure 2A**). Furthermore, after adjusting for other confounding factors, the multivariate survival analysis also showed palliative gastrectomy offers no survival benefit over gastrojejunostomy for symptomatic stage IV GC patients (HR:1.215, 95%CI: 0.859~1.717, *P* = 0.270; **Table 3**, **Supplementary Table 2**). When we further analyzed patients who experienced obstruction, bleeding, or received palliative chemotherapy after surgery, we found that palliative gastrectomy still offers no survival benefit over gastrojejunostomy for symptomatic stage IV GC patients. (**Supplementary Tables 3**-**5**).

**Table 3:** Multivariate survival analysis to compare the long-term survival between palliative gastrectomy and gastrojejunostomy for symptomatic stage IV gastric cancer patients.

Variables	Univariate analysis			Multivariate analysis		
	HR	95% CI	*P-value*	HR	95% CI	*P-value*
All the patients						
Surgical type						
Gastrectomy		Reference			Reference	
Gastrojejunostomy	1.245	[0.917,1.690]	0.160	1.215	[0.859,1.717]	0.270
Multivariate analysis was adjusted for BMI, Tumor location, Symptoms, and Palliative chemotherapy.						
Subgroup-Patients with obstruction						
Surgical type						
Gastrectomy		Reference			Reference	
Gastrojejunostomy	1.147	[0.838,1.570]	0.391	1.195	[0.719,1.988]	0.492
Multivariate analysis was adjusted for BMI, Tumor location, Differentiation, and Palliative chemotherapy.						
Subgroup-Patients with Bleeding						
Surgical type						
Gastrectomy		Reference			Reference	
Gastrojejunostomy	1.012	[0.128,7.971]	0.991	1.539	[0.031,76.256]	0.829
Multivariate analysis was adjusted for BMI, Gender, Tumor location, Differentiation, and Gastric stump cancer.						
Subgroup-Patients received Palliative chemotherapy						
Surgical type						
Gastrectomy		Reference			Reference	
Gastrojejunostomy	1.311	[0.929,1.851]	0.124	1.178	[0.826,1.680]	0.367
Multivariate analysis was adjusted for BMI and Symptoms.						

### 3.4 Survival analysis after PSM

After PSM matching, a total of 144 patients were finally included in this study, 72 in the gastrectomy group and 72 in the gastrojejunostomy group. Baseline characteristics after propensity matching were demonstrated in the **Supplementary Table 6**. The Kaplan–Meier survival curve analysis demonstrated similar prognosis for symptomatic stage IV GC patients who underwent palliative gastrectomy or palliative gastrojejunostomy (**Figure 2B**). However, patients in the gastrectomy group appeared to have a trend toward the best overall survival (*P* = 0.097). Notably, after adjusting for confounding factors, the multivariate survival analysis did not reveal any survival benefit (HR: 0.756, 95%CI: 0.526~1.087, *P* = 0.131) (**Table 4**).

**Table 4:** Univariate and multivariate survival analysis for the stage IV gastric cancer patients who underwent palliative gastrectomy or gastrojejunostomy after PSM.

Variables	Univariate analysis			Multivariate analysis		
	HR	95% CI	*P-value*	HR	95% CI	*P-value*
Age	0.997	[0.981,1.012]	0.669			
BMI	0.938	[0.886,0.998]	0.028	0.931	[0.877,0.987]	0.017
Gender						
Male		Reference				
Female	0.992	[0.690,1.427]	0.966			
Tumor location			0.237			0.238
Proximal		Reference			Reference	
Distal	1.898	[0.882,4.086]	0.101	1.630	[0.749,3.548]	0.218
Total	1.505	[0.475,4.773]	0.487	0.932	[0.279,3.110]	0.908
Smoking history						
Yes						
No	0.985	[0.676,1.434]	0.936			
Drinking history						
Yes		Reference				
No	0.911	[0.614,1.353]	0.645			
Symptoms						
Bleeding		Reference			Reference	
Obstruction	2.770	[0.876,8.762]	0.083	2.716	[0.848,8.699]	0.092
Surgical approach						
Open		Reference				
Laproscope	0.989	[0.500,1.956]	0.974			
Gastric stump cancer						
Yes		Reference				
No	0.984	[0.242,3.996]	0.982			
Linitis plastica						
Yes		Reference				
No	0.919	[0.427,1.977]	0.829			
Differentiation			0.414			
Poor		Reference				
Moderate	0.805	[0.382,1.694]	0.567			
Well	0.695	[0.096,5.038]	0.719			
Unknown	1.271	[0.889,1.817]	0.189			
Lauren classification			0.782			
Intestinal type		Reference				
Diffuse type	1.531	[0.680,3.447]	0.304			
Mixed type	1.423	[0.589,3.443]	0.433			
Unknown	1.407	[0.681,3.910]	0.357			
Surgical type						
Gastrectomy		Reference			Reference	
Gastrojejunostomy	1.336	[0.948,1.883]	0.254	0.756	[0.526,1.087]	0.131
Palliative chemotherapy			0.192			0.191
Yes		Reference			Reference	
No	1.141	[0.419,3.106]	0.797	1.057	[0.381,2.929]	0.915
Unknown	1.669	[0.959,2.904]	0.070	1.745	[0.957,3.181]	0.069

## 4 Discussion

For stage IV gastric cancer patients with obstruction and bleeding, palliative surgery is an important treatment for symptom relief. However, whether palliative gastrectomy or gastrojejunostomy should be conducted was still controversial. In the current study, we found that palliative gastrectomy achieved similar perioperative safety and long-term survival compared with palliative gastrojejunostomy. In addition, same conclusions were obtained regardless of whether we divided the subgroups by different clinical symptoms or tumor locations. These conclusions indicate that, in clinical practice, it is feasible to choose either palliative gastrectomy or gastrojejunostomy for symptomatic stage IV GC patients.

Although NCCN clinical practice guidelines (Version 2.2022) indicate that both palliative gastrectomy and gastrojejunostomy can be reserved for symptomatic stage IV GC patients, there is insufficient evidence to suggest a preference for either surgical option (10). When it comes to choosing between palliative gastrectomy and gastrojejunostomy, the most important thing is the difference in surgical safety and long-term prognosis. Although this issue has been explored in several previous studies, the results are unfortunately controversial (3–7, 11). Most of the previous studies revealed that there was no significant difference in survival between palliative gastrectomy and gastrojejunostomy, although there may have been some heterogeneity in the studies in terms of follow-up time and number of cases (3–6). Similarly, in the current study, we also found that palliative gastrectomy offers no survival benefit for symptomatic stage IV GC patients, despite overall and at the subgroup level. In contrast, a recent multi-center retrospective cohort study conducted in the Japan found that palliative distal gastrectomy might improve the long‐term survival for symptomatic stage IV GC patients (7). We considered that several possible reasons might account for this discrepancy, including the nutritional status, tumor burden, postoperative treatment, and difference in the follow-up time and number of cases.

Firstly, although all the patients included in the studies were stage IV gastric cancer patients with symptoms, the duration of symptoms might cause significant differences in body nutritional status. In fact, a significant proportion of patients made their initial clinical visit after their symptoms have persisted for a long time, especially if they were accompanied by obstruction. Previous study has shown that poor nutritional status before gastrectomy and within 1 year after gastrectomy might lead to poor prognosis (12). For stage IV gastric cancer, more than half of the patients had the European Nutritional Risk Screening (NRS) ≥ 3, which was significantly associated with poor prognosis (13). Thus, despite subsequent palliative surgery, the poor nutritional status might influence the prognosis between the two groups. Secondly, previous studies have shown that patients with high metastatic tumor load also have poor long-term survival using 18F-fluorodeoxyglucose (18F-FDG) positron emission tomography/computed tomography (PET/CT) (14, 15). Given that there may be differences between studies in the site and number of metastases in enrolled GC patients, there may be differences in tumor load among patients, which may lead to differences in long-term prognostic outcomes. Thirdly, it can be found that the acceptance rate of postoperative palliative chemotherapy is relatively low in most studies, and the rate of postoperative palliative chemotherapy is even less than 50% in some studies. However, for patients with advanced gastric cancer, chemotherapy is the most important treatment to improve the prognosis of patients (16). In the present study, the rate of postoperative treatment was 73.5%, which was highest comparing with the previous studies (4, 6, 7, 11). Fourthly, the small number of cases included in previous studies may lack statistical power. In addition, the shorter follow-up period might have substantial effects on outcome differences.

In the present study, we found that although the patients in the palliative gastrojejunostomy group had shorter operation time, and lower proportion of intraoperative blood transfusion, there was no significant difference in postoperative complications. However, in a retrospective study in the Finland demonstrated that palliative gastrectomy group had significantly higher postoperative complications than gastrojejunostomy group (9/26 vs. 2/21) (11). Remarkably, the results of various studies in recent years are similar to ours (4, 7). This may be related to the advances in perioperative management and operative technique. Therefore, palliative gastrectomy has little impact on the perioperative safety compared with gastrojejunostomy.

To the best of our knowledge, the current study was the largest sample size study to discuss the long-term survival between palliative gastrectomy and gastrojejunostomy for symptomatic stage IV GC patients. In our study, the long follow-up period provided more reliable observational outcomes. Nevertheless, we must acknowledge that the present study has some potential limitations. Firstly, this is a dual-center, retrospective, observational study. Secondly, some important clinicopathologic information, including postoperative treatment strategies and preoperative nutritional status were unavailable. Thirdly, During the follow-up, we lacked statistical information on the causes of death, which may have caused some non-neoplastic deaths to affect the outcomes.

## 5 Conclusions

In the current study, we found that palliative gastrectomy provides no survival benefit compared with Gastrojejunostomy for symptomatic stage IV gastric cancer, which indicate that it might be feasible to choose either palliative gastrectomy or gastrojejunostomy for symptomatic stage IV GC patients. However, prospective randomized controlled trials with complete preoperative nutritional status assessment and tumor load assessment are warranted to verify these findings.

## Data Availability

The raw data supporting the conclusions of this article will be made available by the authors, without undue reservation.

## Supplementary material

Supplementary Table 1 Detailed information of metastasis sites of symptomatic stage IV gastric cancer patients who underwent palliative gastrectomy or gastrojejunostomy.

Supplementary Table 2 Univariate and multivariate survival analysis for all the symptomatic stage IV gastric cancer patients who underwent palliative gastrectomy or gastrojejunostomy.

Supplementary Table 3 Univariate and multivariate survival analysis for the stage IV gastric cancer patients with obstructions who underwent palliative gastrectomy or gastrojejunostomy.

Supplementary Table 4 Univariate and multivariate survival analysis for the stage IV gastric cancer patients with bleeding who underwent palliative gastrectomy or gastrojejunostomy.

Supplementary Table 5 Univariate and multivariate survival analysis for the stage IV gastric cancer patients with postoperative palliative chemotherapy who underwent palliative gastrectomy or gastrojejunostomy.

Supplementary Table 6 The baseline characteristics of symptomatic stage IV gastric cancer patients who underwent palliative gastrectomy or gastrojejunostomy after PSM.
