# Supplementary material for: Palliative gastrectomy or gastrojejunostomy for symptomatic stage IV gastric cancer: a dual center, retrospective, propensity matched cohort study
Source: Front Oncol. 2026 Jul 22;16:1892394. doi: 10.3389/fonc.2026.1892394 (PMC13437399; doi:10.3389/fonc.2026.1892394)
Supplement: Supplementary file 1 [file Table1.docx]

**Supplementary Table 1.** Detailed information of metastasis sites of symptomatic stage IV gastric cancer patients who underwent palliative gastrectomy or gastrojejunostomy.

| Metastasis Sites | Total (n=197) | Gastrectomy (n=125) | Gastrojejunostomy (n=72) |
| --- | --- | --- | --- |
| Peritoneal metastasis, n(%) | 145(73.604) | 79(63.200) | 66(91.667) |
| Liver metastasis, n(%) | 33(16.751) | 30(24.000) | 3(4.167) |
| Lung metastasis, n(%) | 5(2.538) | 5(4.000) | 0(0.000) |
| Ovary metastasis, n(%) | 17(8.629) | 16(12.800) | 1(1.389) |
| Non‐regional lymph node metastasis, n(%) | 14(7.107) | 13(10.400) | 1(1.389) |
| Other, n(%) | 3(1.523) | 3(2.400) | 0(0.000) |

**Supplementary Table 2.** Univariate and multivariate survival analysis for all the symptomatic stage IV gastric cancer patients who underwent palliative gastrectomy or gastrojejunostomy.

| Variables | | Univariate analysis | | | Multivariate analysis | | |
| --- | --- | --- | --- | --- | --- | --- | --- |
|  |  | HR | 95% CI | *P-value* | HR | 95% CI | *P-value* |
| Age | | 0.998 | [0.985,1.012] | 0.824 |  |  |  |
| BMI | | 0.959 | [0.914,1.006] | 0.086 | 0.949 | [0.903,0.998] | 0.043 |
| Gender | |  |  |  |  |  |  |
|  | Male |  | Reference |  |  |  |  |
|  | Female | 1.105 | [0.810,1.507] | 0.530 |  |  |  |
| Tumor location | |  |  | 0.267 |  |  | 0.332 |
|  | Proximal |  | Reference |  |  | Reference |  |
|  | Distal | 0.804 | [0.331,1.953] | 0.630 | 1.330 | [0.869,2.035] | 0.189 |
|  | Total | 1.128 | [0.497,2.556] | 0.773 | 0.957 | [0.380,2.409] | 0.926 |
| Smoking history | |  |  |  |  |  |  |
|  | Yes |  | Reference |  |  |  |  |
|  | No | 0.952 | [0.697,1.302] | 0.760 |  |  |  |
| Drinking history | |  |  |  |  |  |  |
|  | Yes |  | Reference |  |  |  |  |
|  | No | 0.986 | [0.714,1.363] | 0.934 |  |  |  |
| Symptoms | |  |  |  |  |  |  |
|  | Bleeding |  | Reference |  |  | Reference |  |
|  | Obstruction | 1.785 | [1.027,3.105] | 0.040 | 1.741 | [0.984,3.080] | 0.057 |
| Surgical approach | |  |  |  |  |  |  |
|  | Open |  | Reference |  |  |  |  |
|  | Laproscope | 1.046 | [0.566,1.933] | 0.885 |  |  |  |
| Gastric stump cancer | |  |  |  |  |  |  |
|  | Yes |  | Reference |  |  |  |  |
|  | No | 0.789 | [0.251,2.478] | 0.685 |  |  |  |
| Linitis plastica | |  |  |  |  |  |  |
|  | Yes |  | Reference |  |  |  |  |
|  | No | 0.85 | [0.472,1.533] | 0.590 |  |  |  |
| Differentiation | |  |  | 0.571 |  |  |  |
|  | Poor |  | Reference |  |  |  |  |
|  | Moderate | 0.757 | [0.438,1.310] | 0.320 |  |  |  |
|  | Well | 0.639 | [0.089,4.594] | 0.656 |  |  |  |
|  | Unknown | 1.103 | [0.809,1.504] | 0.537 |  |  |  |
| Lauren classification | |  |  | 0.551 |  |  |  |
|  | Intestinal type |  | Reference |  |  |  |  |
|  | Diffuse type | 1.387 | [0.774,2.487] | 0.272 |  |  |  |
|  | Mixed type | 0.941 | [0.470,1.885] | 0.864 |  |  |  |
|  | Unknown | 1.184 | [0.708,1.978] | 0.520 |  |  |  |
| Surgical type | |  |  |  |  |  |  |
|  | Gastrectomy |  | Reference |  |  | Reference |  |
|  | Gastrojejunostomy | 1.245 | [0.917,1.690] | 0.160 | 1.215 | [0.859,1.717] | 0.270 |
| Adjuvant treatment | |  |  | 0.446 |  |  | 0.324 |
|  | Yes |  | Reference |  |  | Reference |  |
|  | No | 1.077 | [0.397,2.919] | 0.885 | 0.965 | [0.351,2.659] | 0.946 |
|  | Unknown | 1.252 | [0.885,1.770] | 0.204 | 1.330 | [0.912,1.941] | 0.139 |

**Supplementary Table 3.** Univariate and multivariate survival analysis for the stage IV gastric cancer patients with obstructions who underwent palliative gastrectomy or gastrojejunostomy.

| Variables | | Univariate analysis | | | Multivariate analysis | | |
| --- | --- | --- | --- | --- | --- | --- | --- |
|  |  | HR | 95% CI | *P-value* | HR | 95% CI | *P-value* |
| Age | | 1.001 | [0.987,1.016] | 0.860 |  |  |  |
| BMI | | 0.938 | [0.892,0.987] | 0.013 | 0.934 | [0.886,0.985] | 0.012 |
| Gender | |  |  |  |  |  |  |
|  | Male |  | Reference |  |  |  |  |
|  | Female | 0.935 | [0.677,1.291] | 0.683 |  |  |  |
| Tumor location | |  |  | 0.368 |  |  | 0.563 |
|  | Proximal |  | Reference |  |  | Reference |  |
|  | Distal | 1.352 | [0.879,2.080] | 0.170 | 1.148 | [0.719,1.834] | 0.564 |
|  | Total | 1.108 | [0.422,2.906] | 0.835 | 0.741 | [0.267,2.054] | 0.564 |
| Smoking history | |  |  |  |  |  |  |
|  | Yes |  | Reference |  |  |  |  |
|  | No | 0.857 | [0.619,1.185] | 0.350 |  |  |  |
| Drinking history | |  |  |  |  |  |  |
|  | Yes |  | Reference |  |  |  |  |
|  | No | 0.851 | [0.605,1.196] | 0.353 |  |  |  |
| Surgical approach | |  |  |  |  |  |  |
|  | Open |  | Reference |  |  |  |  |
|  | Laproscope | 0.999 | [0.525,1.901] | 0.998 |  |  |  |
| Gastric stump cancer | |  |  |  |  |  |  |
|  | Yes |  | Reference |  |  |  |  |
|  | No | 1.058 | [0.261,4.285] | 0.937 |  |  |  |
| Linitis plastica | |  |  |  |  |  |  |
|  | Yes |  | Reference |  |  |  |  |
|  | No | 0.886 | [0.479,1.639] | 0.700 |  |  |  |
| Differentiation | |  |  | 0.360 |  |  | 0.380 |
|  | Poor |  | Reference |  |  | Reference |  |
|  | Moderate | 0.626 | [0.353,1.108] | 0.108 | 0.595 | [0.323,1.096] | 0.096 |
|  | Well | 0.581 | [0.081,4.185] | 0.590 | 0.570 | [0.075,4.348] | 0.587 |
|  | Unknown | 1.026 | [0.742,1.419] | 0.877 | 0.971 | [0.578,1.631] | 0.912 |
| Lauren classification | |  |  | 0.824 |  |  |  |
|  | Intestinal type |  | Reference |  |  |  |  |
|  | Diffuse type | 1.187 | [0.656,2.147] | 0.571 |  |  |  |
|  | Mixed type | 0.890 | [0.444,1.784] | 0.743 |  |  |  |
|  | Unknown | 1.060 | [0.631,1.783] | 0.825 |  |  |  |
| Surgical type | |  |  |  |  |  |  |
|  | Gastrectomy |  | Reference |  |  | Reference |  |
|  | Gastrojejunostomy | 1.147 | [0.838,1.570] | 0.391 | 1.195 | [0.719,1.988] | 0.492 |
| Adjuvant treatment | |  |  | 0.533 |  |  | 0.330 |
|  | Yes |  | Reference |  |  | Reference |  |
|  | No | 1.038 | [0.382,2.819] | 0.942 | 1.148 | [0.405,3.251] | 0.795 |
|  | Unknown | 1.229 | [0.857,1.762] | 0.262 | 1.373 | [0.904,1.086] | 0.137 |

**Supplementary Table 4.** Univariate and multivariate survival analysis for the stage IV gastric cancer patients with bleeding who underwent palliative gastrectomy or gastrojejunostomy.

| Variables | | Univariate analysis | | | Multivariate analysis | | |
| --- | --- | --- | --- | --- | --- | --- | --- |
|  |  | HR | 95% CI | *P-value* | HR | 95% CI | *P-value* |
| Age | | 0.983 | [0.942,1.026] | 0.430 |  |  |  |
| BMI | | 1.170 | [0.954,1.435] | 0.132 | 1.498 | [1.031,2.177] | 0.034 |
| Gender | |  |  |  |  |  |  |
|  | Male |  | Reference |  |  | Reference |  |
|  | Female | 9.069 | [2.063,39.872] | 0.004 | 16.529 | [1.353,201.981] | 0.028 |
| Tumor location | |  |  | 0.644 |  |  |  |
|  | Proximal |  | Reference |  |  |  |  |
|  | Distal | 2.070 | [0.446,9.611] | 0.353 |  |  |  |
|  | Total | 2.127 | [0.185,24.461] | 0.545 |  |  |  |
| Smoking history | |  |  |  |  |  |  |
|  | Yes |  | Reference |  |  |  |  |
|  | No | 2.010 | [0.614,6.578] | 0.249 |  |  |  |
| Drinking history | |  |  |  |  |  |  |
|  | Yes |  | Reference |  |  |  |  |
|  | No | 2.010 | [0.614,6.578] | 0.249 |  |  |  |
| Surgical approach | |  |  |  |  |  |  |
|  | Open |  | Reference |  |  |  |  |
|  | Laproscope | 1.436 | [0.179,11.543] | 0.734 |  |  |  |
| Gastric stump cancer | |  |  |  |  |  |  |
|  | Yes |  | Reference |  |  | Reference |  |
|  | No | 0.208 | [0.022,2.000] | 0.174 | 0.087 | [0.003,2.581] | 0.158 |
| Linitis plastica | |  |  |  |  |  |  |
|  | Yes |  | Reference |  |  |  |  |
|  | No | 0.832 | [0.105,6.108] | 0.862 |  |  |  |
| Differentiation | |  |  | 0.149 |  |  | 0.086 |
|  | Poor |  | Reference |  |  | Reference |  |
|  | Moderate | 14.890 | [0.916,242.085] | 0.058 | 277.748 | [0.777,99274.751] | 0.061 |
|  | Well |  |  |  |  |  |  |
|  | Unknown | 1.234 | [0.226,3.187] | 0.809 | 1.373 | [0.034,56.136] | 0.867 |
| Lauren classification | |  |  | nan |  |  |  |
|  | Intestinal type |  |  |  |  |  |  |
|  | Diffuse type |  |  |  |  |  |  |
|  | Mixed type |  |  |  |  |  |  |
|  | Unknown |  |  |  |  |  |  |
| Surgical type | |  |  |  |  |  |  |
|  | Gastrectomy |  | Reference |  |  | Reference |  |
|  | Gastrojejunostomy | 1.012 | [0.128,7.971] | 0.991 | 1.539 | [0.031,76.256] | 0.829 |
| Adjuvant treatment | |  |  | 0.533 |  |  |  |
|  | Yes |  | Reference |  |  |  |  |
|  | No |  |  |  |  |  |  |
|  | Unknown | 1.759 | [0.451,6.859] | 0.416 |  |  |  |

**Supplementary Table 5.** Univariate and multivariate survival analysis for the stage IV gastric cancer patients with postoperative chemotherapy who underwent palliative gastrectomy or gastrojejunostomy.

| Variables | | Univariate analysis | | | | Multivariate analysis | | |
| --- | --- | --- | --- | --- | --- | --- | --- | --- |
|  |  | HR | 95% CI | *P-value* | HR | | 95% CI | *P-value* |
| Age | | 0.992 | [0.997,1.008] | 0.325 |  | |  |  |
| BMI | | 0.953 | [0.903,1.006] | 0.082 | 0.941 | | [0.890,0.995] | 0.032 |
| Gender | |  |  |  |  | |  |  |
|  | Male |  | Reference |  |  | |  |  |
|  | Female | 1.022 | [0.706,1.479] | 0.910 |  | |  |  |
| Tumor location | |  |  | 0.492 |  | |  |  |
|  | Proximal |  | Reference |  |  | |  |  |
|  | Distal | 1.314 | [0.813,2.125] | 0.265 |  | |  |  |
|  | Total | 1.019 | [0.347,2.996] | 0.973 |  | |  |  |
| Smoking history | |  |  |  |  | |  |  |
|  | Yes |  | Reference |  |  | |  |  |
|  | No | 0.895 | [0.621,1.289] | 0.550 |  | |  |  |
| Drinking history | |  |  |  |  | |  |  |
|  | Yes |  | Reference |  |  | |  |  |
|  | No | 0.881 | [0.604,1.286] | 0.512 |  | |  |  |
| Symptoms | |  |  |  |  | |  |  |
|  | Bleeding |  | Reference |  |  | | Reference |  |
|  | Obstruction | 1.830 | [0.982,3.411] | 0.057 | 1.926 | | [1.005,3.690] | 0.048 |
| Surgical approach | |  |  |  |  | |  |  |
|  | Open |  | Reference |  |  | |  |  |
|  | Laproscope | 0.890 | [0.433,1.827] | 0.750 |  | |  |  |
| Gastric stump cancer | |  |  |  |  | |  |  |
|  | Yes |  | Reference |  |  | |  |  |
|  | No | 0.722 | [0.229,2.276] | 0.578 |  | |  |  |
| Linitis plastica | |  |  |  |  | |  |  |
|  | Yes |  | Reference |  |  | |  |  |
|  | No | 0.938 | [0.412,2.135] | 0.879 |  | |  |  |
| Differentiation | |  |  | 0.502 |  | |  |  |
|  | Poor |  | Reference |  |  | |  |  |
|  | Moderate | 1.125 | [0.554,2.286] | 0.744 |  | |  |  |
|  | Well | 0.760 | [0.105,5.515] | 0.786 |  | |  |  |
|  | Unknown | 1.310 | [0.915,1.875] | 0.141 |  | |  |  |
| Lauren classification | |  |  | 0.840 |  | |  |  |
|  | Intestinal type |  | Reference |  |  | |  |  |
|  | Diffuse type | 1.066 | [0.504,2.256] | 0.867 |  | |  |  |
|  | Mixed type | 0.731 | [0.287,1.862] | 0.512 |  | |  |  |
|  | Unknown | 0.963 | [0.500,1.855] | 0.910 |  | |  |  |
| Surgical type | |  |  |  |  | |  |  |
|  | Gastrectomy |  | Reference |  |  | | Reference |  |
|  | Gastrojejunostomy | 1.311 | [0.929,1.851] | 0.124 | 1.178 | | [0.826,1.680] | 0.367 |

**Supplementary Table 6.** The baseline characteristics of symptomatic stage IV gastric cancer patients who underwent palliative gastrectomy or gastrojejunostomy after PSM.

| Variables | | Total (n=144) | Gastrectomy (n=72) | Gastrojejunostomy (n=72) | *P*-value |
| --- | --- | --- | --- | --- | --- |
| Age (mean±SD） | | 55.340±11.397 | 53.917±11.454 | 56.764±11.160 | 0.136 |
| BMI (mean±SD) | | 22.680±3.394 | 22.783±3.395 | 22.570±3.389 | 0.713 |
| Gender, n(%) | |  |  |  | 0.034 |
|  | Male | 96(66.667) | 42(58.333) | 54(75.000) |  |
|  | Female | 48(33.333) | 30(41.667) | 18(25.000) |  |
| Tumor location, n(%) | |  |  |  | 1.000 |
|  | Proximal | 10(6.944) | 5(6.944) | 5(6.944) |  |
|  | Distal | 128(88.889) | 64(88.889) | 64(88.889) |  |
|  | Total | 6(4.167) | 3(4.167) | 3(4.167) |  |
| Smoking history, n(%) | |  |  |  | 0.846 |
|  | Yes | 35(24.306) | 17(23.611) | 18(25.000) |  |
|  | No | 109(75.694) | 55(76.389) | 54(75.000) |  |
| Drinking history, n(%) | |  |  |  | 0.848 |
|  | Yes | 37(26.812) | 19(27.536) | 18(26.087) |  |
|  | No | 101(73.188) | 50(72.464) | 51(73.913) |  |
| Symptoms, n(%) | |  |  |  | 1.000 |
|  | Bleeding | 6(4.167) | 3(4.167) | 3(4.167) |  |
|  | Obstruction | 138(95.833) | 69(95.833) | 69(95.833) |  |
| Surgical approach, n(%) | |  |  |  | 0.049 |
|  | Open | 134(93.056) | 64(88.889) | 70(97.222) |  |
|  | Laproscope | 10(6.944) | 8(11.111) | 2(2.778) |  |
| Surgical margins, n(%) | |  |  |  | nan |
|  | Negative | 68(94.444) | 68(94.444) | nan |  |
|  | Positive | 4(5.556) | 4(5.556) | nan |  |
| Gastric stump cancer, n(%) | |  |  |  | 1.000 |
|  | Yes | 2(1.389) | 1(1.389) | 1(1.389) |  |
|  | No | 142(98.611) | 71(98.611) | 71(98.611) |  |
| Linitis plastica, n(%) | |  |  |  | 0.145 |
|  | Yes | 7(4.861) | 5(6.944) | 2(2.778) |  |
|  | No | 137(95.139) | 67(93.056) | 70(97.222) |  |
| Differentiation, n(%) | |  |  |  | nan |
|  | Poor | 63(43.750) | 56(77.778) | 7(9.722) |  |
|  | Moderate | 8(5.556) | 7(9.722) | 1(1.389) |  |
|  | Well | 1(0.694) | 0(0.000) | 1(1.389) |  |
|  | Unknown | 72(50.000) | 9(12.500) | 63(87.500) |  |
| Lauren classification, n(%) | |  |  |  | <0.001 |
|  | Intestinal type | 11(8.661) | 10(15.385) | 1(1.613) |  |
|  | Diffuse type | 23(18.110) | 20(30.769) | 3(4.839) |  |
|  | Mixed type | 10(7.874) | 6(9.231) | 4(6.452) |  |
|  | Unknown |  |  |  |  |
| Adjuvant treatment, n(%) | |  |  |  | 0.628 |
|  | Yes | 124(86.111) | 60(83.333) | 64(88.889) |  |
|  | No | 5(3.472) | 3(4.167) | 2(2.778) |  |
|  | Unknown | 15(10.417) | 9(12.500) | 6(8.333) |  |
